# Spectral Weighting Underlies Perceived Sound Elevation

**DOI:** 10.1038/s41598-018-37537-z

**Published:** 2019-02-07

**Authors:** Bahram Zonooz, Elahe Arani, Konrad P. Körding, P. A. T. Remco Aalbers, Tansu Celikel, A. John Van Opstal

**Affiliations:** 1 Biophysics Department, Donders Institute for Brain, Cognition, and Behaviour, Radboud University, 6525 AJ Nijmegen, The Netherlands; 2 Department of Bioengineering, University of Pennsylvania, Philadelphia, PA USA; 3 Department of Neuroscience, University of Pennsylvania, Philadelphia, PA USA; 4 Neurophysiology Department, Donders Institute for Brain, Cognition, and Behaviour, Radboud University, 6525 AJ Nijmegen, The Netherlands

## Abstract

The brain estimates the two-dimensional direction of sounds from the pressure-induced displacements of the eardrums. Accurate localization along the horizontal plane (azimuth angle) is enabled by binaural difference cues in timing and intensity. Localization along the vertical plane (elevation angle), including frontal and rear directions, relies on spectral cues made possible by the elevation dependent filtering in the idiosyncratic pinna cavities. However, the problem of extracting elevation from the sensory input is ill-posed, since the spectrum results from a convolution between source spectrum and the particular head-related transfer function (HRTF) associated with the source elevation, which are both unknown to the system. It is not clear how the auditory system deals with this problem, or which implicit assumptions it makes about source spectra. By varying the spectral contrast of broadband sounds around the 6–9 kHz band, which falls within the human pinna’s most prominent elevation-related spectral notch, we here suggest that the auditory system performs a weighted spectral analysis across different frequency bands to estimate source elevation. We explain our results by a model, in which the auditory system weighs the different spectral bands, and compares the convolved weighted sensory spectrum with stored information about its own HRTFs, and spatial prior assumptions.

## Introduction

A crucial stage in the neural processing for sensory perception involves source localization. When the sensory space is encoded topographically, like in touch and vision, stimulus location is encoded as spatial maps, and dynamically updated by efference copies of intervening movements. Sensorimotor integration across these dynamic spatial representations enables accurate somatosensory and visual localization performance^1–3^.

The auditory system, however, is tonotopically organized at the level of the cochlea, and has to derive the stimulus location and its properties from pressure-induced implicit acoustic cues at the eardrums. For source localization in the horizontal (azimuth) plane, the system takes advantage of binaural acoustic difference cues, by decoding position from temporal and intensity differences across the ears^4^. However, as all sources in the midsagittal plane, and on the so-called’ inter-aural cone of confusion’, yield identical binaural differences, these cues are not sufficient to uniquely resolve the 2D direction of a sound. To resolve the cone of confusion, the auditory system should have access to the sound’s elevation angle in the vertical plane as well.

**Figure 1:**
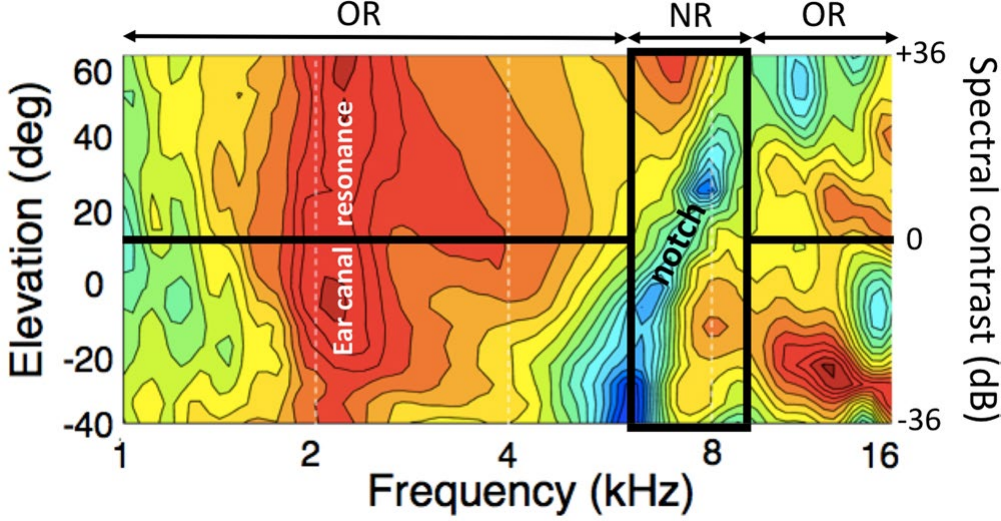
Color-coded HRTFs (amplitude spectra (1–16 kHz) between −15 dB (dark blue) and +20 dB (dark red)) as function of elevation for a typical human subject, with our high-contrast stimuli overlaid in black lines. NR: notch region (6–9 kHz); OR: outer region. Note the prominent elevation-dependent spectral notch (blue). Also, between 4–6 kHz spectral cues vary monotonically with elevation, albeit less strongly than in the NR. The cues above 9 kHz vary in a much more complex way with elevation. At the low frequencies, the ear-canal resonance around 2.5 kHz (red) is direction-independent.

It is well established that the elevation of a sound in vertical planes is derived from pinna-related spectral-shape cues^5–12^, which are described by elevation-specific and idiosyncratic Head-Related Transfer Functions (HRTFs; Fig. 1)^5,6,11–15^. The acoustics underlying the spectral cues that encode sound-source elevation are not yet fully understood, but to a first approximation they can be related to the superposition of direct and reflected high-frequency (>3–4 kHz) sound waves in the ear canal. As the pinna is asymmetrically shaped, the pinna reflections will follow different path lengths, depending on the incident angle and particular geometry of the pinna cavities and ridges, leading to constructive and destructive interference at specific frequencies that differ for each direction in elevation (^11^, and see^14^ for an elaborate 3D model).

Importantly, the problem to extract the sound’s elevation from the acoustic spectrum, say at ε^∗^, is mathematically ill-posed. The sensory input at the eardrums, *s*(*t*; ε^∗^), results from a time-domain convolution between the actual source pressure, *x*(*t*), and the impulse response of the particular HRTF associated with the source’s elevation, *h*(*t*; ε^∗^), both of which are a-priori unknown to the auditory system^6,7^. For the frequency representation in the cochlea, the sensory spectrum is given by *S*(*f*; ε^∗^) = *H*(*f*; ε^∗^)·*X*(*f*), yielding one equation with two unknowns (*H* and *X*). As a result, the extraction of source elevation (and hence the veridical 2D sound direction) cannot be performed with certainty, as there exists no unique solution. Still, despite this fundamental problem, human sound-localization performance can be very accurate and precise in all directions^6,7,13^, even during fast eye-head movements^16^.

To estimate the most likely elevation angle, i.e., the true *H*(*f*; ε^∗^), and thus to successfully disambiguate the cone of confusion, the auditory system should also incorporate non-acoustic information about sounds and the environment. It is not clear how the auditory system accomplishes this task, or what implicit assumptions it makes about potential sound-source spectra in natural habitats. For example, the brain could consider certain parts of the sensory information to be more reliable than others, and hence put different weights on different frequency bands. Conversely, it could exclusively process only those parts of the spectrum that yield an optimal spatial resolution.

Previous models have suggested that to estimate the sound’s elevation, the human auditory system might perform a cross-correlation between incoming and learned spectral HRTF patterns^6–8,10,11,15,17^, calculate a spectrally weighted average (Covert Peak Area^18–21^), or rely on a comparison of the input with elevation-specific local spectral derivatives^22,23^. The HRTF analysis has been shown to integrate the spectral information from the two ears^24^, and to include frequencies exceeding 8 kHz^25^. Yet, regardless of their differences in signal processing, these models all somehow compare the current sensory spectrum to stored features of HRTF templates. They thus assume prior perceptual learning for reliable source localization performance. Several studies have indeed demonstrated that the human auditory system successfully adapts to chronic changes in its HRTFs^26–30^.

In this paper, we studied perceptual mislocalizations to systematic manipulations of the acoustic spectral input, in order to unravel the underlying neural strategies of the auditory system. Based on the above, we reasoned that source (mis)localization in elevation should depend systematically on the spectral properties of the stimulus, in particular on those parts of the spectrum that encode spatial information either more, or less, reliably. We thus varied spectral contrast by changing the relative power over a ±36 dB range within a restricted, but relevant, portion of the major pinna-induced spectral notch (from 6–9 kHz for the human ear^13,14,22,23^; see Fig. 1), with respect to the surrounding frequency bands (1–6 kHz and 9–20 kHz).

Importantly, however, also these surrounding bands contain elevation-specific information, albeit less reliable. For example, as can be seen in Fig. 1, the HRTFs vary systematically and monotonically with elevation between 4–6 kHz, but their changes are markedly smaller than for the 6–9 kHz band. On the other hand, the cues above 9 kHz seem to be strong, but are more erratic (non-monotonic). In addition, hearing performance at those high frequencies is relatively poor. Hence, if the auditory system would rely on the spectral information across the entire acoustic spectrum, some localization performance might still be possible, even when all cues from the 6–9 kHz band were removed. On the other hand, if only specific features (like raising spectral edges)^22,23^ from the 6–9 kHz band would contribute to the elevation percept, for being the strongest and most reliable sensory input, localization performance would be affected, but remain invariant to changes in the contribution of surrounding bands. However, if the auditory system would weigh the different frequency bands differently, e.g. depending on their reliability, localization is expected to systematically depend on the relative power provided by the different frequency bands.

A recent study provided some supporting evidence for this latter possibility^31^. When listeners were confronted with low-pass filtered sounds, containing only poor spectral cues (cut-off at 6 kHz; Fig. 1), the stimulus-response relation in elevation was typically characterised by a low gain (slope), and a considerable upward response bias. However, repeated exposure of these sounds at a limited number of locations on the midsagittal plane induced a gradual improvement of their responses, even without any sensory feedback. The response gain increased, and the bias and mean-squared localization errors were reduced, also for other spectral stimuli, and for different locations across frontal space. As the HRTFs (including their spectral derivatives, or covert peak areas) had not been tampered with, we interpreted this result as evidence for an increased contribution of the low-frequency HRTF bands to the elevation estimate^31^.

To further test this idea, we here measured open-loop sound-localization performance of normal-hearing subjects over a wide range of azimuth and elevation directions in the frontal hemifield, while manipulating the relative acoustic power in the surrounding bands and notch band. Our results indicate a systematic influence of spectral contrast between these bands on the stimulus-response relationships. While spectral contrast modulated the elevation gain in a non-monotonic and asymmetric way, we also found that extra power within the notch band introduced a large upward localization bias, and sometimes up-down confusions. We extended the spectral cross-correlation model for the elevation localization system^7^ to account for these results.

## Results

To investigate the relative contributions of the different frequency bands to the localization percept, we measured 2D sound- localization performance of eight normal-hearing listeners (see Materials and Methods), while systematically varying the acoustic power within the Notch Region (NRI) with respect to the Outer Region of the broadband spectrum (ORI; Fig. 1), and overall sound level.

**Figure 2:**
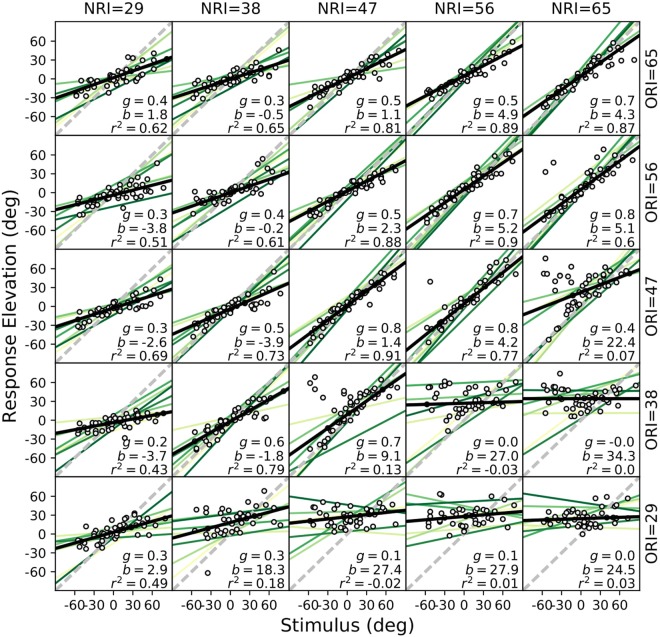
Elevation results for all stimuli from listener P1 (circles: observed data; solid black line: optimal regression). Green lines: regressions for the other seven subjects. Elevation performance varies strongly with spectral contrast, and with the absolute stimulus power. Even in the absence of power in the notch band (upper-left section), response elevation correlates with stimulus elevation (*r*^*2*^ ∼ 0.6; *g* ∼ 0.4). The response bias increased markedly for high positive contrasts (lower-right section). Outliers at the smaller positive contrasts in the lower-right section of the figure, indicate up-down confusions. Note that all listeners showed qualitatively similar response patterns.

**Figure 3:**
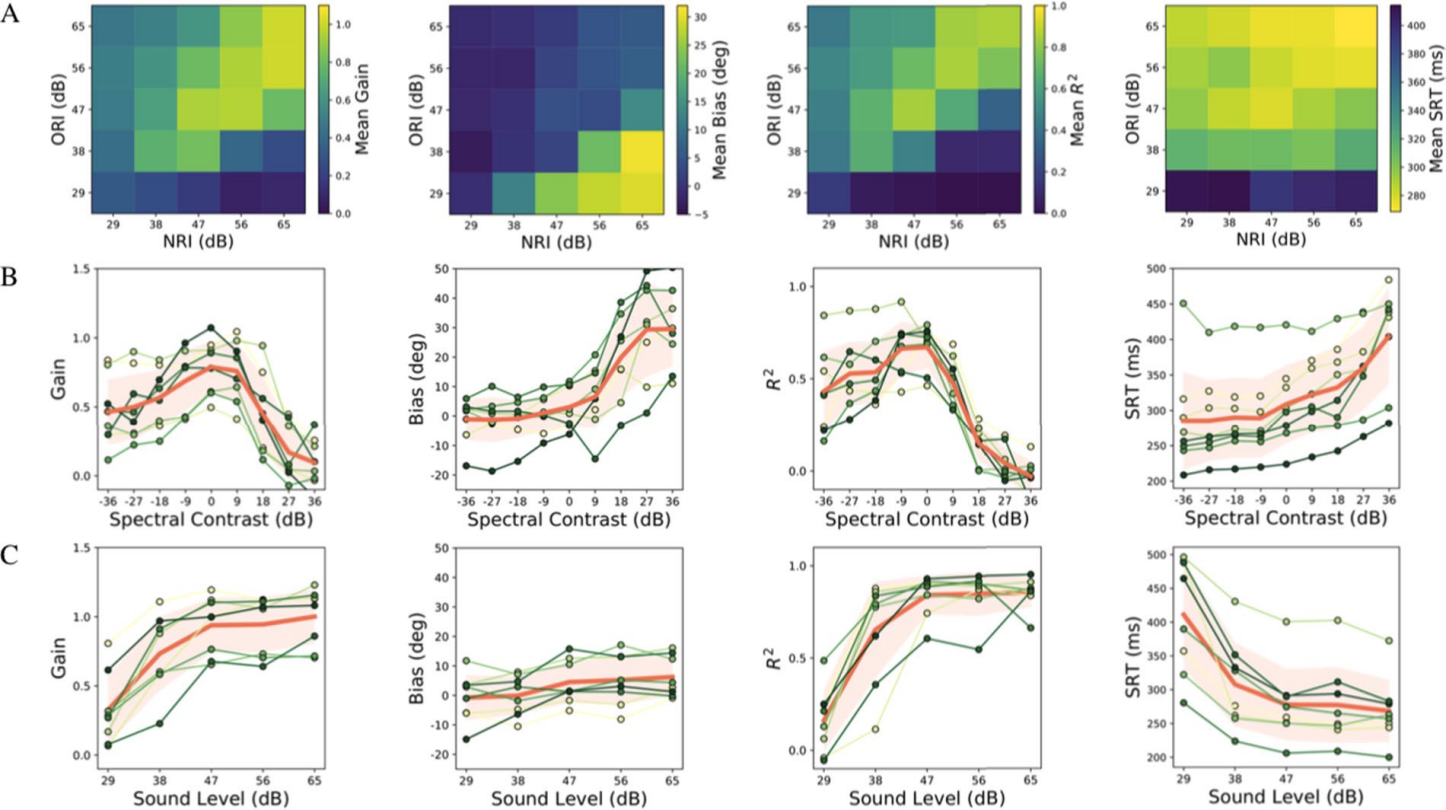
(**A**) Average effect of NRI and ORI, (**B**) influence of spectral contrast (given by NRI-ORI, i.e. along the anti-diagonals), and (**C**) influence of sound level (along the main diagonal) on elevation gain (left column), elevation bias (second column), localization precision (*r*^2^) (third column), and reaction times (right column), averaged across listeners. The thick solid orange line and shaded orange area indicate mean and standard deviation, respectively. Green lines: results from individual participants. Note that elevation gain and precision were maximal at near-zero contrast, and that the non-monotonic dependence on contrast was asymmetric. For high positive contrasts, the response gain and precision rapidly approached zero, while the upward bias rapidly increased. The response reaction times increased systematically with increasing (positive) spectral contrast, and with decreasing sound level.

The localization performance of listener P1 for the elevation response components to all 25 spectral stimuli is shown in Fig. 2 (symbols, and black lines). The regression results from the other seven participants are also indicated (green lines). The spectral manipulations had a strong effect on the listener’s elevation percepts. The response gain (i.e., accuracy) and the coefficient of determination (*r*^2^; a measure of response precision) between stimulus location and response degraded systematically with decreasing ORI levels (data plots along columns) but varied non-monotonically with decreasing NRI levels (along rows). Even in the absence of acoustic power in the notch region, response elevation was still correlated with stimulus elevation (*r* ∼ 0.8), albeit at a lower gain (*g*∼0.3–0.4; upper-left) and more variability (*r*^2^~0.6). Further, the response bias increased to ∼+30 deg for large positive spectral contrasts, at very low response gains for all subjects (*g* ≈ 0; lower-right). At smaller positive contrasts one may note the occurrence of occasional up-down confusions (seen as outliers; see Discussion). Although the individual results differed quantitatively, the qualitative response patterns were quite similar across subjects (Fig. 3). Thus, although most information about elevation is typically found in the HRTF notch region (Fig. 1), a veridical elevation percept requires a much broader acoustic spectrum, whereby the different spectral regions appear to contribute in different ways to the system’s elevation estimate.

In line with earlier reports (e.g.^32^), localization in the vertical plane was sensitive to the signal-to-noise ratio (SNR), as the perceived elevation varied more strongly with stimulus intensity than perceived azimuth (Fig. 2, main diagonal of the stimulus matrix, and Supplementary Information). When the stimulus was easily detectable (≥47 dB, which is 17 dB above the laboratory’s background noise), a further increase in sound level, without changing spectral contrast, did not further improve localization performance (compare NRI = ORI = [47 56 65] dB). As the SNR decreased, localization performance deteriorated accordingly (compare NRI = ORI = [29 38] dB).

In contrast to the elevation components, results for the azimuth response components were accurate and precise for all subjects (Fig. S2 in Supplementary information), and nearly independent of the intensity of the notch and outer frequency bands. Only at the lowest sound levels and highest positive contrasts (lower-right and lower-left of the stimulus matrix) one may observe a slight deterioration of the response precision, as evidenced from the increased variability (lower *r*2; Fig. S2).

### Gain and precision

To quantify the interaction of spectral contrast (defined as NRI-ORI) and stimulus level, we performed regression analyses across the 25 stimulus combinations of NRI and ORI for each subject, and calculated the spatial gains and biases, as well as *r*^2^, for the goodness of fit (Fig. 3A). If subjects had responded accurately, regardless of NRI, ORI, and overall sound level, uniform patterns should have emerged (all yellow for gain and *r*^2^, all blue for the offsets). Instead, the elevation results systematically depended on spectral contrast and on overall sound levels. High accuracy, low response bias, and high fidelity were obtained for stimuli with the lowest spectral contrasts (stimuli near and on the main diagonal of the matrix), and at the highest sound levels (top-right corners of the stimulus matrix). Reduction of overall sound level along the main diagonal of the stimulus matrix systematically reduced accuracy and precision of perceived sound elevation.

### Bias

The localization bias was largely independent of stimulus intensity across the different spectra (Fig. 3C, center), but it increased sharply for high positive spectral contrasts for all listeners (Fig. 3B, center). This means that excessive acoustic power within the notch region typically induced a strong upward elevation percept. At moderate positive spectral contrasts, however, subjects sometimes perceived up-down confusions, which showed up as a bistable response behavior (e.g., Fig. 2 for P1).

### Reaction times

Assuming that responses are initiated faster at high confidence levels for source location^7^, we analyzed the head-saccade reaction times for all stimuli and subjects (Fig. 3, right-hand column). Head movements were initiated systematically earlier at high sound levels, and low spectral contrasts. Attenuation of the NRI and/or ORI band systematically prolonged the reaction times. When the stimulus intensity approached the background noise floor, irrespective of spectral contrast (bottom row of the stimulus matrix), responses were delayed the most (compare ORI = [29 38] across NRI). In contrast, absolute NRI level did not significantly affect the reaction times (columns), although with increased ORI levels subjects progressively responded earlier (rows).

## Discussion

We studied the effect of manipulating spectral contrast with the central notch band (6–9 kHz) on 2D sound localization behavior. Our experiments set out to dissociate different hypotheses regarding spectral-cue analysis in the auditory system, in particular, how and whether different spectral bands contribute to the perceived elevation of sound sources. As the most prominent elevation-dependent spectral cues of human pinnae typically lie within the 5–10 kHz band, we systematically manipulated spectral contrast of broadband sounds in the 6–9 kHz band with the surrounding frequency bands (0.5–6 kHz and 9–20 kHz) over a ±36 dB range. We deliberately did not remove or enhance the entire notch band between 5–10 kHz. Our stimuli were constructed such that also the surrounding frequency bands would still contain some systematic, albeit weaker and therefore less reliable, elevation-related spectral cues (see the example HRTFs of Fig. 1).

The experiments could thus allow us to assess the relative contributions from different spectral regions to perceived elevation. We reasoned that if the auditory system would indeed include weighted spectral information from outside the major spectral notch region, listeners would be able to localize sound elevation, even in the absence of the spectral notch, albeit at a different response gain, depending on the reliability of the cues. In addition, when spectral power would be exclusively confined to the spectral notch region, for which the cues are considered highly reliable and strong, localization performance might still deteriorate when compared to full broadband sounds without (much) contrast.

Alternatively, if the auditory system would solely rely on the spectral information from the central notch region, our spectral manipulations would result in only minor effects on sound-localization performance.

In contrast to this latter expectation, all subjects systematically mislocalized source elevation when spectral contrast was highly positive (Figs 2 and 3), and also when all cues from the central notch region were removed. Best localization performance (high gain, low bias, and little variability) was obtained for low-contrast stimuli (nearly flat GWN), in which all spectral cues would be present.

We also observed an interesting asymmetry in the gains and variability measures as function of spectral contrast (Fig. 3B): performance to sounds containing only the surrounding bands (at *C* <−18 dB) was much better (response gains between 0.4–0.5, bias close to zero, *r*^2^ > 0.5) than localization to high positive-contrast sounds with attenuated spectral surrounds (at *C* >+ 18 dB). Subjects consistently perceived these latter sounds at a fixed upward elevation (gains and *r*^2^ close to zero, bias around +30 deg, although the absolute bias values varied considerably from subject to subject).

Note that stimuli with high positive spectral contrast (lower-right in the stimulus matrix) could induce a sensory spectrum, *S*(*f*; ε), that best resembles the HRTFs for upward locations (cf. Fig. 1), as even for the low elevations, for which the spectral notch is the only dominant cue, the high stimulus power may exceed the suppressive influence of the notch. As a result, the net stimulus spectrum will preserve a positive peak within the central notch region for *all* source elevations. At the more moderate contrasts, however, this competition could lead to up-down confusions, due to noisy fluctuations in the sensory processing chain, for which either the spectral peak (upward bias response), or the notch will win (veridical elevation response; see Fig. 2). Stimuli with large negative spectral contrasts, however, will preserve (and even emphasize) the spectral notch within the notch band, but nonetheless still contain some systematic elevation-dependent spectral cues in the 4–6 kHz band of the outer region across all elevation directions. These latter surviving cues, however, are less prominent and, therefore, less reliable than those in the notch region. We hypothesise that this particular property may underlie the observed significant stimulus-response relation, but at a reduced response gain.

In summary, we conjecture that these results are in line with a weighted spectral comparison across the entire acoustic spectrum of the sensory input with stored knowledge about the system’s own HRTFs, where the reliability of the different spectral cues modulates the gain of the stimulus-response relation. A mechanism that readily accounts for such behavior is based on Bayesian inference (see Supplementary Information).

### Models underlying the elevation percept

Two major types of models have been forwarded in the literature to explain the extraction of the elevation angle from the spectral cues: feature-based models vs. wide-band correlative models. Despite their differences, all models rely on some internal comparison between the incoming convolved source spectrum and stored information (or neural representation) about either the particular spectral features, or about the entire HRTF. Moreover, all models require a decision mechanism that determines which of the comparisons with the stored templates yields the most likely elevation estimate. The main difference between the feature-based and wide-band spectral models is that the spectral analysis in the former is confined to narrow bands (typically up to one octave bandwidth^23^), rather than to the full relevant spectrum between 3.5–15 kHz.

### Feature models

Feature-based models emphasise particular aspects of the HRTFs, like the elevation-dependent location of a peak (the so-called covert-peak area^18–21^), or a notch-based analysis, which has been proposed to quantify spectral contrast by taking the spectral derivatives^22^.

The spectral derivatives idea^22^ was recently refined by Baumgartner and colleagues^23^. Their raising-edge model extracts only the maxima of the positive flanks within the major spectral notch of the HRTFs. Their model^23^ was inspired by single-unit recordings from the cat’s dorsal cochlear nucleus (DCN). The DCN contains spectral-sensitive cells that have been hypothesized to form a computational circuit^33,34^ that extracts the positive spectral edges from the cat’s HRTFs, and could thus play a major role in the cat’s sound-localisation behaviour^34,35^. Perhaps, a similar mechanism could exist in the human auditory system, although the spectral features of human HRTFs differ from those of cats^36^.

### Wide-band models

Several psychophysical studies have suggested that the extraction of the elevation angle could involve an analysis that concerns the entire relevant HRTF spectrum^6,12,15^. For example, by presenting stimuli with randomly rippled amplitude spectra over the 2.5–16 kHz band, with the peaks and valleys varying over ±20 dB, listeners would often perceive idiosyncratic illusionary source elevations. These illusions depended reliably on the particular source spectrum and occurred despite the fact that the listener knew that the speaker remained at a fixed, straight-ahead location. By applying linear maximum-likelihood estimation on the stimulus-response distributions, an elevation-dependent spectral function could be reconstructed that would represent the major spectral features underlying the listener’s elevation percepts (the posterior spectral distribution function^11,12^). Interestingly, these spectral reconstructions had a remarkable resemblance to the full idiosyncratic DTFs of the listener, rather than to a particular notch or peak as perceptual spectral feature.

In a recent study we showed that listeners improved the accuracy of their goal-directed head-movements to low-pass filtered noises (bandwidth from [0.5–6 kHz]), repeatedly presented at only six different locations in the mid-sagittal plane^31^. Importantly, the improvements occurred without any sensory feedback (i.e., no visual information, and stimulus durations were 150 ms, which is much shorter than the typical head-movement reaction time), and generalized to stimuli with other spectral properties (like notch-band sounds) elicited across the two-dimensional frontal space. Such behaviour is not immediately expected from a spectral feature model without any spectral weighting^23^, as the HRTFs themselves were not affected by these sounds, and any spectral features present would remain unaltered throughout the experiment. Instead, those results suggest that the behavioural changes reflected an increased weighting of the low-frequency spectral band in the HRTFs (e.g. between 3.5–6 kHz), where elevation-specific information is present (at least in larger pinnae^28^; e.g. Fig. 1), albeit relatively weak, and less robust against noise than the 6–9 kHz band.

In what follows, we describe a wide-band neurocomputational model that attempts to capture the major experimental findings of the present study, as summarised in Fig. 3. The model incorporates the statistical inference framework that is supposed to underlie many aspects of sensory perception, and of sensory-motor integration. It extends an earlier proposal from our group^7^, in which we described an explicit cross-correlation model that is robust to a wide variety of spectral stimulus shapes, but also predicts the particular spatial illusions for stimuli with certain non-flat spectral shapes.

### Computational model

**Figure 4:**
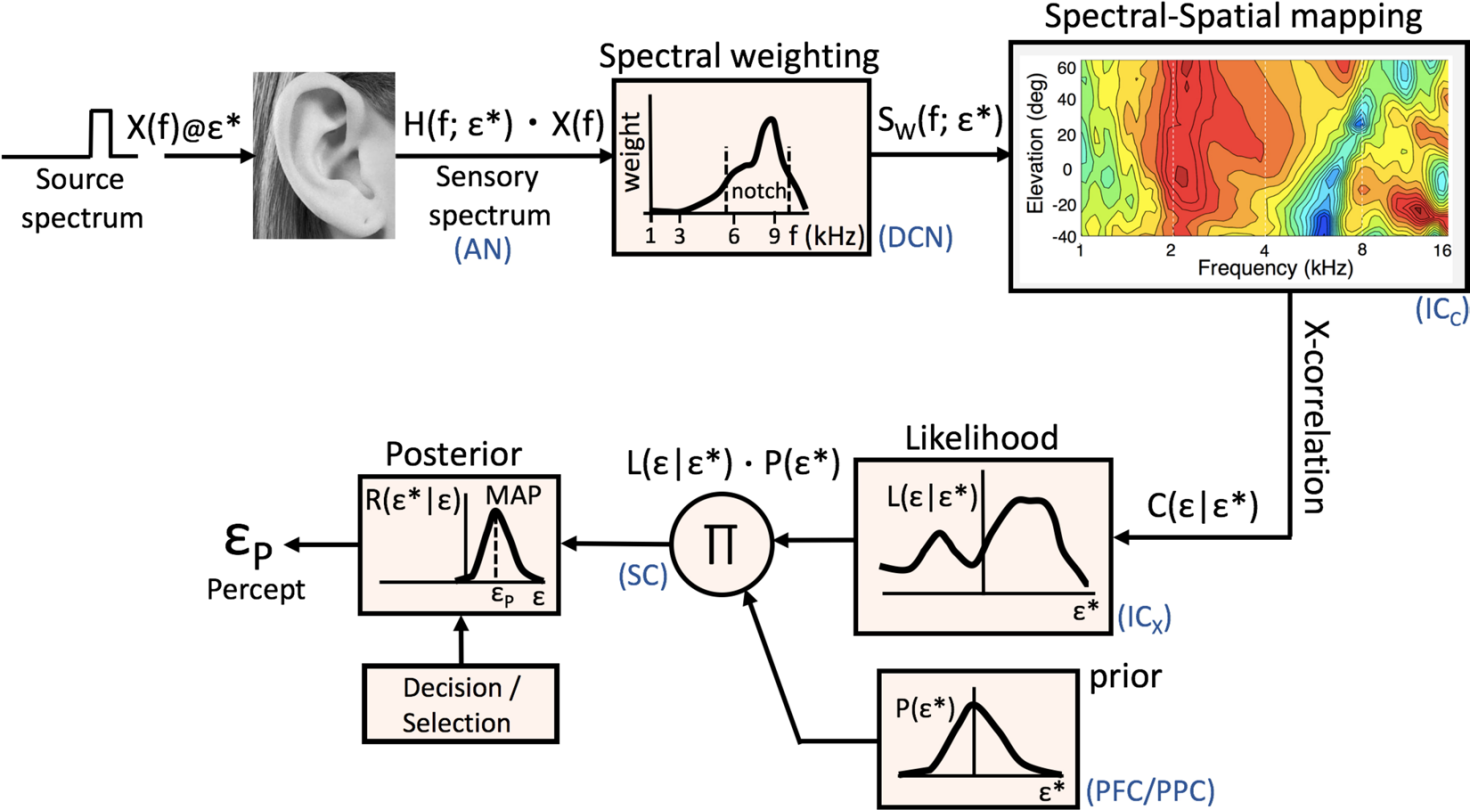
Generative model explaining how the input spectrum results in an elevation percept. The sensory spectrum is a convolution of the sound with the HRTF of its associated elevation, ε^∗^. Different frequency bands around the notch area are weighted differently, depending on the strength and reliability of their spatial information content, resulting in a weighted sensory spectrum, *S*_*W*_(*f*, ε^∗^). The latter is cross-correlated with stored information about all HRTFs, resulting, after rectification, in a likelihood distribution of potential source locations, *L*(ε|ε^∗^). Subsequently, Bayesian inference weighs the sensory evidence against its internal prior (taken around straight ahead), resulting in a more precise posterior. Finally, the decision stage selects the maximum of the posterior (MAP), to provide an optimal estimate for the perceived elevation angle, ε_P_. Blue labels indicate putative neural stages, described in the text.

In the presence of noise, optimal decisions should be based on a trade-off between the (noisy) sensory evidence and (non-acoustic) information about expected stimulus properties and locations. The latter may be derived from previous experience and is represented by prior distributions. Results from humans and animals across a broad range of sensorimotor conditions, have been successfully modeled with the Bayesian framework, e.g.,^37–42^. Our neuro-computational model, shown in Fig. 4, can account for the results of Figs 2 and 3. The model is based on a spectral cross-correlation stage proposed earlier by others and by our group^6,7,11,12^. According to that idea, the auditory system compares (i.e., cross-correlates) the current sensory input spectrum, *S*(*f*; ε^∗^), with stored (presumably) full representations of all HRTFs, while relying on two prior internal assumptions: (i) the HRTFs are unique for each elevation angle (i.e. they do not correlate with each other), and (ii) source spectra, *X*(*f*), do not correlate with any of the stored HRTFs. We have shown that if both conditions are met that the maximum of the correlation function is always found at the veridical source elevation. In this way, the system could successfully cope with the ill-posed nature of the localisation problem described in the Introduction for a wide variety of spectral stimuli^6,7,15^.

The current proposal extends this model in several ways: first, instead of weighting the entire spectral input homogeneously, it imposes a weighting function on the spectral input, which attenuates particular spectral bands according to their spatial information content and to their reliability. Second, the output of the cross-correlation stage between the weighted spectral input and the stored HRTFs, is rectified in order to represent a likelihood function of potential source elevations, *L*(ε|ε^∗^). Third, the sensory likelihood is combined with internal priors regarding potential source locations, *P*(ε^∗^), which describe the system’s (learned) expectations about the environment. The subsequent multiplication of the likelihood with the spatial prior then results in a posterior estimate of potential target locations that gave rise to the sensory input: *R*(ε^∗^|ε) ∝ *L*(ε|ε^∗^)·*P*(ε^∗^). In the final decision stage, the system makes an optimal (or near-optimal^43^) decision on the basis of the posterior distribution. For Gaussian distributions, the decision is optimal when it selects the maximum of the posterior, as the responses then have the smallest averaged squared error at the smallest variability. This decision strategy is known as the maximum-a-posteriori (or MAP) estimate.

The extensions to the original spectral cross-correlation model can account for the observed average behavior of the response gain, bias and variability, shown in Fig. 3 (see Supplementary Information, for details). It is important to note that we did not attempt to fit the individual results of each subject, shown in Figs 2 and 3, as we were mainly interested in explaining the overall response patterns. These resulted to be quite consistent across subjects (Fig. 3), despite the individual differences around the mean. Moreover, quantitative differences in the behavioural results could be due to several factors, related to the acoustic, non-acoustic, and neural processing chain (see below), and would require an extensive parameter search in order to fit the model. We regard such an exercise beyond the scope of the present paper.

To illustrate the potential of the model, we generated a set of artificial HRTFs that resembled the recorded data of Fig. 1 (see Fig. S4 and Supplemental Material). For the spectral weighting function we took a function that attained nonzero values only between 2.5–12 kHz, with a single peak around 8 kHz (i.e., in the center of the notch band). For frequencies below 5–6 kHz the weighting had low values, to express the relatively weak changes of the spectral cues with elevation. For frequencies beyond 9 kHz the weights were low too, to reflect the poor sensitivity of the human auditory system at these high frequencies.

**Figure 5:**
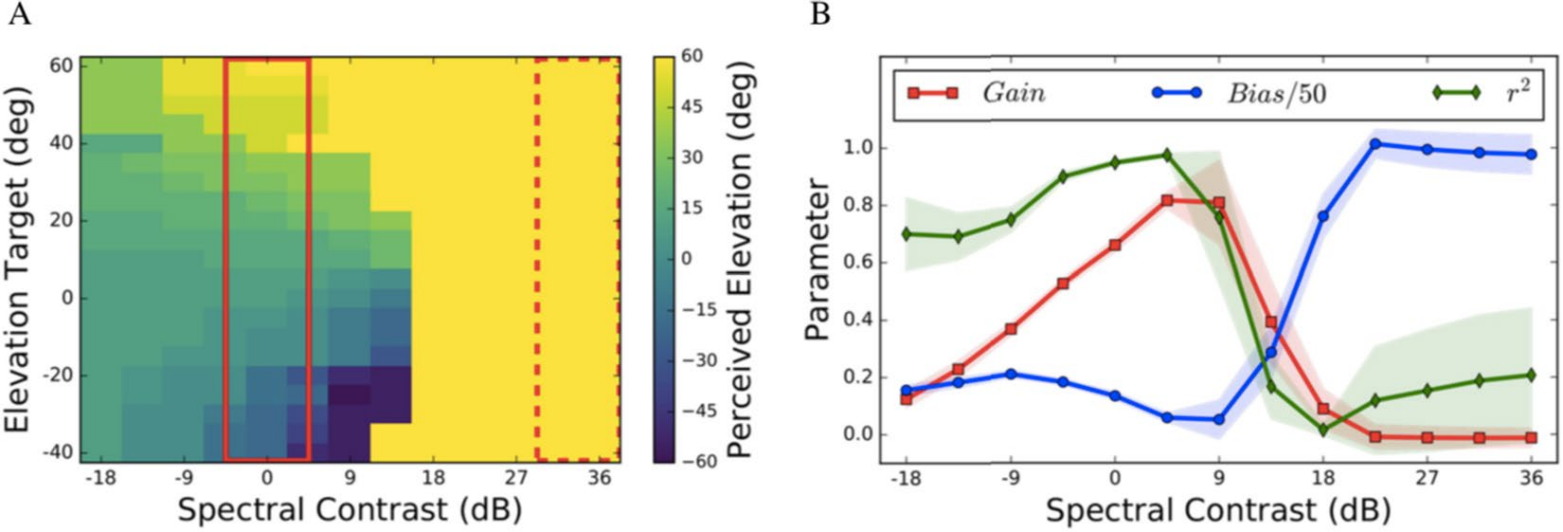
Simulation results of the model (Fig. 4) using a parameterized approximation of HRTFs (Fig. S4A), and a spectral weighting function with α_1_ = 4.5, α_2_ = 1.5 (see Methods, and Fig. S4B). (**A**) Perceived elevation is color-coded for different spectral contrasts, and actual source elevations. Solid red box: results for flat noise (zero contrast). Dashed red box: result for high positive contrast, yielding strong upward biases. Note also the presence of up-down confusions at moderate positive contrasts (at around +12 db). (**B**) The simulations agree qualitatively with the experimental data of Fig. 3B. Red line: response accuracy (gain) as function of contrast is asymmetrically shaped; blue: response bias (note different scale; in deg); green: response precision (*r*^*2*^).

Figure 5 shows that the model can indeed capture the essential patterns observed in the average data: an asymmetric dependence of the response gain and response variability on spectral contrast, and a steep increase of the response bias at high positive contrasts. Note that a spatial prior (e.g. that all targets are confined to the frontal hemifield) may alleviate potential front-back confusions, which are often observed in experiments using sounds with poor spectral properties. In our experiments we never obtained such front-back confusions, Yet, as discussed above, some positive contrast spectra led to up-down confusions.

### Inter-subject variability

Although the stimulus-response patterns for the spectral contrast stimuli were qualitatively similar across subjects, quantitative variability was apparent too (Fig. 3). Several factors may have contributed to these idiosyncratic differences in results: (i) the spectral weighting function could be under adaptive control, and depend, e.g., on the use of sensorimotor feedback, or the current acoustic environment^31^; (ii) the HRTFs themselves are known to be highly idiosyncratic; (iii) the spatial prior distribution will depend on the individual’s active interaction with sounds in her environment, on task constraints, pre-knowledge, etc., and will likely differ from subject to subject too; (iv) the decision function may be task dependent as well (e.g., optimal MAP estimation, or other sub-optimal decision strategies on the posterior, like random sampling^43^). Different types of experiments than performed in this paper would be needed to address and dissociate these different factors, which falls beyond the scope of this study.

### Neural mechanisms

Several neural stages in the audio-motor system could potentially correspond to the different mechanisms described in our model. As the auditory nerve (AN) faithfully represents the sensory input, the HRTF spectrum could be mediated already at an early stage, presumably at the dorsal cochlear nucleus (DCN)^33–35^. We here speculate that the spectral weighting stage could thus be implemented as early as the DCN-to-auditory-midbrain projection. As several studies have implicated a prominent role for the inferior colliculus (IC) in spatial auditory processing^44–46^ its central nucleus (ICc) might embed the cross-correlation stage, with the recruited population in its external nucleus (ICx) representing the likelihood function. Because the ICx projects directly to the midbrain superior colliculus (SC), the localized Gaussian population in the SC motor map could correspond to the posterior distribution of potential eye-head orienting responses^47^. Its output would thus reflect the final selection and decision stage of the model^48^. Learned spatial prior information could descend as a top-down signal from higher auditory centers (e.g., prefrontal (PFC) and posterior parietal (PPC) cortices) in order to modulate the input to the SC population.

## Methods

### Participants

Eight listeners (P1-P8, ages 19–30; 6 males, 2 females) participated in the free-field sound localization experiments. Seven listeners were naive as to the purpose of the study, and were familiarized with the experimental set-up and localization paradigm prior to the start of the experiments. None of the participants reported any hearing impairments or use of a restorative hearing aid.

### Ethics statement

The local Ethics Committee of the Faculty of Social Sciences of the Radboud University (ECSW) approved the experimental procedures (ECSW2016-2208-41). All experiments adhered to the relevant guidelines and regulations for which ethical approval was obtained. All subjects gave their full written informed consent prior to their participation.

### Experimental setup

Experiments were conducted in a dark, sound-attenuated echo-free room (3.2 × 3.2 × 3.5 m), with a background noise level of 30 dB SPL (A-weighted). All four walls, ceiling, floor and every large object present, were covered with sound-attenuating foam (50 mm thick with 30 mm pyramids, AX2250, Uxem b.v., Lelystad, The Netherlands). The listener’s head was in the center of the room at a minimum distance of 1.6 m from the walls. Acoustic measurements (with Brüel and Kjaer BK2610 amplifier, and Brüel and Kjaer BK4144 microphone) at different positions in the room, showed a slight reverberation only for low frequencies (around 500 Hz) near the walls of the room. We verified that the listener’s ears were within the room’s reverberation radius (the critical distance) for the low-frequency stimuli (approximately 1.1 m at T60 = 0.09 s, given that the absorption coefficient of the walls for 500 Hz sounds was about 0.7; taken from the manufacturer’s data sheet). From this, we conclude that the listeners were exposed to the loudspeaker’s direct sound field only, without any appreciable reverberations.

Subjects sat in a chair, positioned at the center of a vertically-oriented circular hoop (1.2 m radius) on which 58 small broad-range loudspeakers *(Visaton SC5.9, Haan, Germany)* were mounted, allowing for a 2.5 deg resolution in elevation on the midsagittal plane between −60 and +85 deg. The hoop could rotate around the vertical axis, yielding an azimuth resolution <0.1 deg. The sound produced by the motor did not provide any cue to the subject about the upcoming sound location, neither in azimuth, nor in elevation. To prevent the potential cue of movement duration of the motor, a random movement of at least 20 deg was always interspersed in the inter-trial interval before directing the hoop to the desired azimuth angle. In earlier control experiments in our lab the absence of any knowledge about the hoop’s location had been verified by letting subjects guess the position of the hoop in darkness when no sound had been presented from one of the speakers. This yielded zero correlation between guessed responses and actual hoop locations.

The speakers had a nearly flat response characteristic between 0.02–20 kHz: fluctuations in their amplitude characteristics remained within ±3 dB between 200 and 3000 Hz, and within ±2 dB across the high end of the spectrum >3 kHz, which were not corrected for in the stimulus generation. For examples of transfer characteristics of these speakers, we refer the reader to the manufacturer’s website at http://www.visaton.com/en/industrie/breitband/sc5_9nd_8.html.

At the center of each speaker, a single light-emitting diode (LED) could serve as a visual target. The LED (size approximately 5 mm) did not disturb the sound field emanating from the speaker cone directly behind it.

Target locations of the speakers on the hoop and head movement responses were transformed to double-pole (azimuth-elevation) coordinates^49^. Azimuth, α, is defined as the angle between the sound source and the midsagittal plane. Elevation, ε, is taken as the angle between the line from the center of the head to the target, referenced to the horizontal plane through the center of the head. The origin of the coordinate system corresponded to the straight-ahead speaker location. Note that in this coordinate system, the sum of the absolute values of the azimuth and elevation angles can never exceed 90 deg: |α| + |ε| ≤ π/2.

Head movements were recorded with the magnetic search-coil technique^50^. To that end, the participant wore a lightweight (150 g) “helmet” consisting of two perpendicular 4 cm wide straps that could be adjusted to fit around the participant’s head without interfering with the ears. On top of this helmet, a small induction coil was attached. From the left side of the helmet, a 40 cm long, thin, aluminum rod protruded forward with a dim (0.15 Cd/m^2^) red LED attached to its end, which was positioned in front of the subject’s eyes and served as a head-fixed and eye-fixed pointer. In this way, we ensured that subjects always pointed to the perceived location with their eyes, while generating a full gaze-shift head movement. Two orthogonal pairs of 3.2 × 3.2 m coils were attached to the edges of the room to generate the horizontal (60 kHz) and vertical (80 kHz) oscillating magnetic fields, required for this method. The head-coil signals were amplified and demodulated (Remmel Labs, Ashland, MA), low-pass filtered at 150 Hz (custom-made, 4th-order Butterworth), and stored on hard disk at a sampling rate of 500 Hz per channel for off-line analysis.

### Sound generation

Sounds were digitally generated using Tucker-Davis Technologies (TDT) (Alachua, FL) System III hardware, with a TDT DA1 16-bit digital-to-analog converter (48,828.125 Hz sampling rate). A TDT PA4 programmable attenuator controlled sound level, after which the stimuli were passed to the TDT HB6 buffer and finally to one of the speakers in the experimental room. All 25 spectral contrast sounds were derived from a common GWN signal of 150 ms duration, sampled at the TDT A/D - D/A sampling frequency of 48,828.125 Hz. The band-stop and band-pass signals were generated by applying a zero phase digital filter (using Matlab’s function filtfilt.m from the Signal Processing Toolbox), with the target band between 6–9 kHz. The steepness of the filter in the pass- or stop band was 120 dB/octave. This signal was passed to the TDT system-3 D/A hardware to be delivered to the speakers at the required, calibrated, sound levels. Example spectra of the two extreme notch-filtered sounds are shown in the Supplementary Information, Fig. S1.

**Figure 6:**
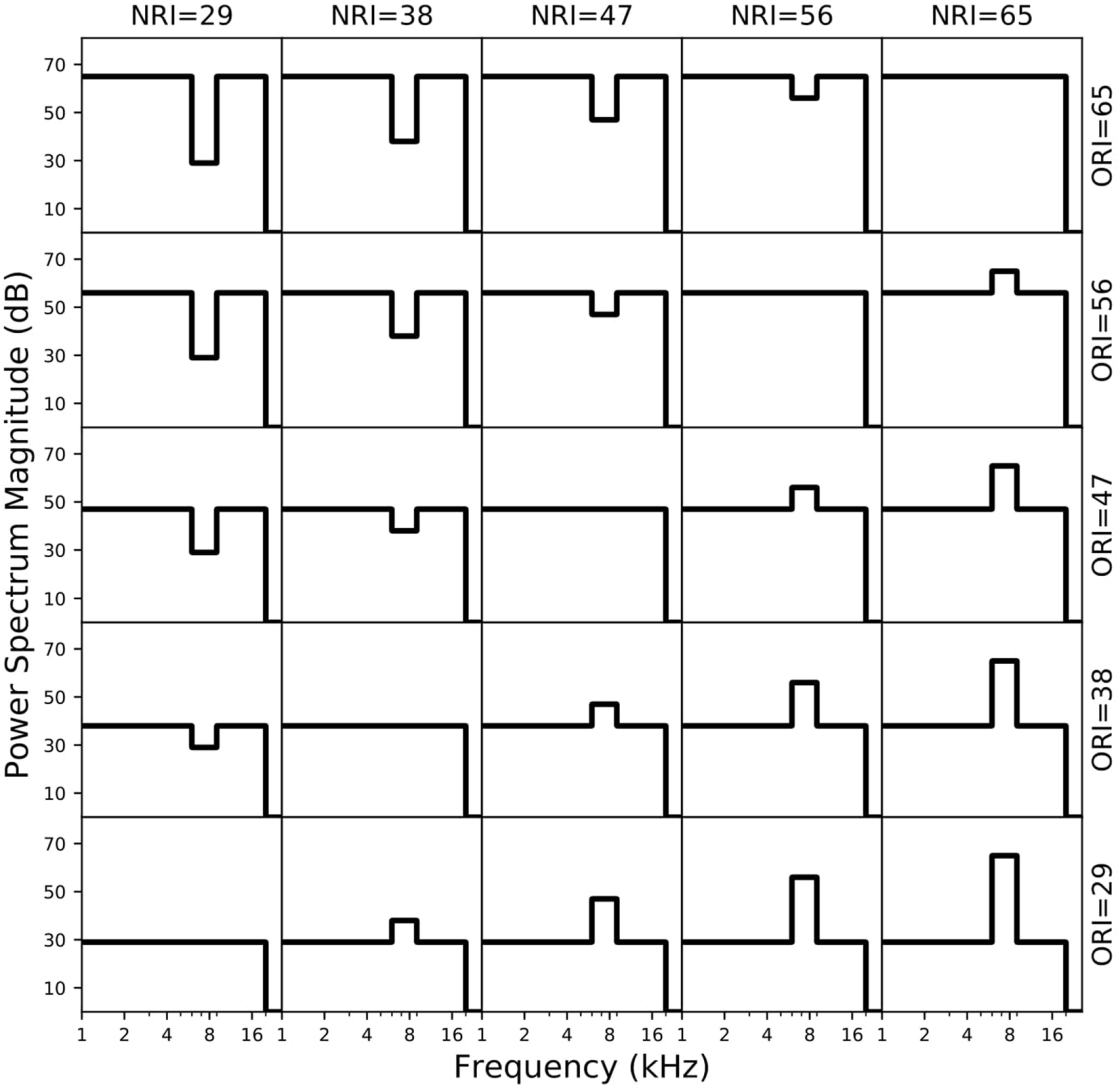
Schematic power spectra of the 25 sounds in the experiment. The notch region was confined to 6–9 kHz, and the outer regions contained the 0.5–6 kHz and 9–20 kHz bands (see Supplementary Information, for details). Note that the lowest sound level (29 dB SPL) was close to the background noise level of the laboratory room (∼30 dBA).

All acoustic stimuli were derived from a 150 ms Gaussian white noise signal with a flat spectrum between 0.5–20 kHz, with 5 ms sine-squared onset and cosine-squared offset ramps. Twenty-five different auditory stimuli were used in the experiment, which differed in their spectral amplitudes within and outside of the 6–9 kHz notch band. Sounds were created by varying the intensity of the notch band (Notch Region Intensity, or NRI) and the intensity of the remaining spectral bands (Outer Region Intensity, or ORI: 0.5 < *f* < 6 kHz and 9 < *f* < 20 kHz). The default control stimulus was flat Gaussian White Noise (GWN) (top-right in Fig. 6). A stimulus is defined by its NRI and ORI, which both were varied in the range of 29, 38, 47, 56, and 65 dB. Each sound was presented once from forty-eight positions during the experiment, randomly selected from α ∈ [−90, 90] deg, and ε ∈ [−60,85] deg in the frontal hemifield. Absolute free-field sound levels had been measured at the position of the subject’s head with a calibrated sound amplifier and microphone (Brüel and Kjaer, BK2610 and BK4144, respectively).

### Experimental paradigm

Each experimental session started with a calibration to establish the mapping of coil signals to known target locations. Head-position data for the calibration procedure were obtained by instructing the subject to make an accurate head movement while redirecting the dim red LED in front of the eyes from the central green fixation LED to each of 58 peripheral LEDs, illuminated as soon as the fixation point extinguished. The 58 fixation points and raw head-position signals were used to train two three-layer neural networks that served to calibrate the head movement data, using the Bayesian regularization implementation of the back-propagation algorithm (MatLab; Neural Networks Toolbox).

In the sound-localisation experiments, a trial started by fixating the central LED. After this LED extinguished, an auditory target was presented 400 msec later at a pseudo-randomly selected location. The subject was asked to quickly redirect the head by pointing the dim red LED on the helmet (see Supplementary Information) to the perceived location of the sound stimulus^2^. The experiment contained 1200 trials per subject: 25 stimuli at 48 randomly selected azimuth-elevation directions, divided into 8 blocks of 150 trials. If the target was perceived at a rear location, the instruction was to point the dim LED at the mirror-imaged location in the frontal field, and to press the hand-held button. In this way, the experimenter could correct the response off-line as a rear response by: ε_rear_ = 180-ε_front_^12^. In none of the current experiments, however, subjects reported rear percepts.

### Data Analysis

A custom-written Matlab script automatically detected head saccades in the calibrated data by using a preset velocity criterion (15^◦^/*s*) for head-movement onset and offset^6^. We fitted the responses, for each participant and stimulus type, with linear regression lines: α_*R*_ = *g*_1_ · α_*T*_ + *b*_1_ and ε_*R*_ = *g*_2_ · ε_*T*_ + *b*_2_, respectively. α_*R*_ and ε_*R*_ are the azimuth and elevation response components, and α_*T*_ and ε_*T*_ are the actual azimuth and elevation coordinates of the target. Fit parameters, *b*_1_ and *b*_2_, are the biases (offsets; in degrees), whereas *g*_1_ and *g*_2_ are the gains (slopes, dimensionless) of the azimuth and elevation responses, respectively. To find optimal fit parameters, we minimized the mean absolute error, which is less sensitive to potential outliers in the data^51^. From the regressions, we also calculated Pearson’s linear correlation coefficient, *r*, the coefficient of determination, *r*^2^, the residual error (SD around the fitted line), and the mean absolute localization error.

### Model implementation

The generative model of Fig. 4 was implemented in Matlab (version 15Ra; for code, see Supplemental Material) and consisted of the following steps: First, the sensory spectrum, *S*(*f*), was determined by adding the sound spectrum, *X*(*f*) (in dB; Fig. 6) to the amplitude spectrum (in dB) of the associated HRTF (see Fig. S4) at stimulus location ε^∗^. The multiplication of spectra from the linear time-convolution converts to spectral summation, because of the logarithmic cochlear representation, resulting from the compressive nature of the nonlinear cochlear amplifier (outer hair cells, signal transmission from inner hair cells to auditory nerve). In this way, sound levels become expressed on dB scale at the cochlear output stage. A first novel feature of the extended model, compared to^6,11^, is the inclusion of a non-monotonic spectral weighting function, *w*(*f*), which leads to a weighted sensory spectrum, *S*_*W*_(*f*; ε^∗^):

$${S}_{W}(f;\varepsilon \ast )=w(f)\cdot (H(f;\varepsilon \ast )+X(f))$$

The underlying idea is that the auditory system has learned to optimally employ the potential spectral localization cues in the sensory input for further spatial analysis. Cues that are deemed more reliable (strong, consistent) obtain a stronger weight than cues that are considered less reliable (weak, noisy).

The weighted sensory spectrum is subsequently cross-correlated with all (stored) HRTFs. This cross-correlation is performed over the relevant spectral bands in the signal (3–12 kHz). It results in a function of ε and ε^∗^: *C*(ε|ε^∗^), which will contain peaks at those elevations, ε^∗^ (i.e., HRTFs) that most resemble the weighted sensory spectrum, caused by *X*(*f*) at ε^∗^.

The second new feature in our model is the likelihood function, *L*(ε|ε^∗^), which is obtained from rectifying the correlation function. In other words, only positive correlations could potentially relate to the stimulus location.

The third new component in the model involves a Bayesian inference stage, which combines the likelihood function with a prior distribution of expected source locations (we here assumed a Gaussian with zero mean and a standard deviation, σ_*prior*_), in combination with a decision and selection stage on the resulting posterior. Optimal inference, in the sense of the smallest absolute error at the lowest variability, would take the maximum of the posterior to specify the response (the MAP decision rule). However, different decision mechanisms could be at play (e.g. taking a random sample from the posterior; this would be suboptimal, but was recently proposed as a possible strategy for localization in the elevation direction^43^).

In our simulations we generated a canonical set of HRTFs that resembled the data in Fig. 1. The remaining three free parameters of the model were: the shape of the spectral weighting function, *w*(*f*) (here confined to 2 parameters), and the width, σ_*P*_, of the prior distribution. In our simulations (Fig. 5), we took [*f*_*min*_, *f*_*max*_] = [3.5, 12] kHz, and thus modeled the spectral weighting function by a negatively skewed beta function for which the lower end of the spectrum (3–6 kHz) and high end of the spectrum (9–12 kHz) attained low values:

$$w(f)=a\cdot {(f-fmin)}^{{\alpha }_{1}}\cdot {(fmax-f)}^{{\alpha }_{2}}for\,fmin < f < fmax$$

with α_2 < _α_1_. This heuristic function rises to a peak in the center of the notch band, and rapidly decays to zero for *f*_*max*_ = 12 kHz, as at the highest frequencies, the spectral cues in the HRTFs become unreliable because of the high hearing thresholds. For simplicity, we further took a near-uniform, broad prior distribution, for which *P*(ε^∗^) ≈ *constant* across the range of elevation angles.

## Supplementary information

Supplementary Information
