# Supplementary material for: Spectral Weighting Underlies Perceived Sound Elevation
Source: Sci Rep. 2019 Feb 7;9:1642. doi: 10.1038/s41598-018-37537-z (PMC6367479; doi:10.1038/s41598-018-37537-z)
Supplement: Supplementary file 1 — Supplementary Information [file 41598_2018_37537_MOESM1_ESM.pdf]

Supplementary Information

Spectral Weighting Underlies Perceived Sound Elevation

Bahram Zonooz[1], Elahe Arani[1], Konrad P. Körding[2,3], P.A.T. Remco Aalbers[1], Tansu Celikel[4], and A. John Van Opstal[1,*]

[1] Biophysics Department, Donders Institute for Brain, Cognition, and Behaviour, Radboud University, 6525 AJ, Nijmegen, The Netherlands

[2] Department of Bioengineering, University of Pennsylvania, Philadelphia, PA, USA

[3] Department of Neuroscience, University of Pennsylvania, Philadelphia, PA, USA

[4] Neurophysiology Department, Donders Institute for Brain, Cognition, and Behaviour, Radboud University, 6525 AJ, Nijmegen, The Netherlands

[*]Corresponding author: j.vanopstal@donders.ru.nl

1. Stimulus spectra. Figure S1 provides two examples of stimulus power spectra (calibrated in dBA) as used in the experiments. The spectra were calculated from the white-noise samples generated in Matlab; the solid black line shows a windowed ongoing average through the data. The stimuli correspond to the top-left and bottom-right sounds of Figure 6, respectively.

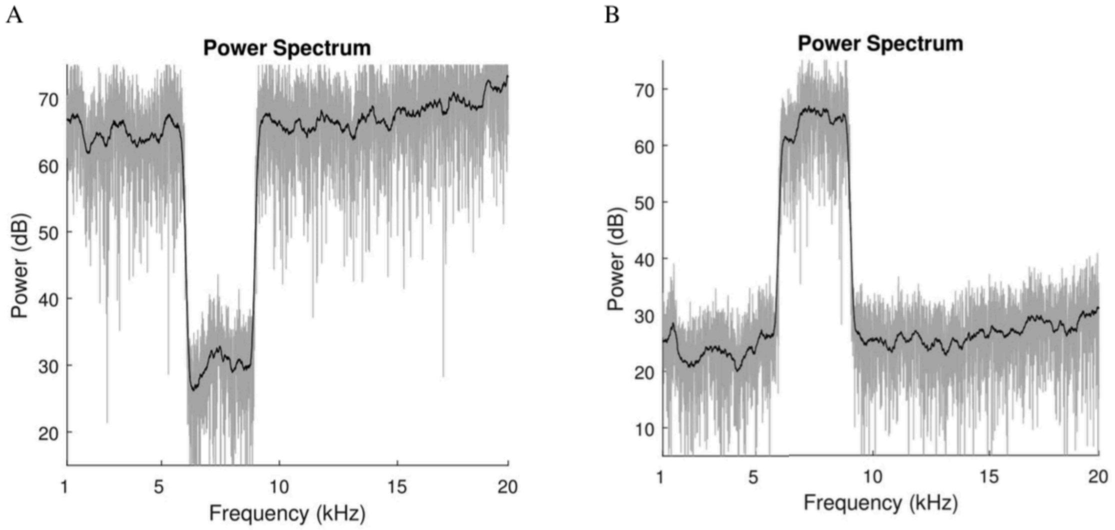

Figure S1. The calculated power spectra for a bandstop (at -36 dB) (A), and a bandpass (at +36 dB) (B) acoustic stimulus, as used in the experiments. The band extends from 6-9 kHz.

2. Azimuth response components. In Figure S2 we present the influence of spectral contrast and stimulus level on the azimuth response components of all subjects. The azimuth localization for different values of NRI and ORI for the 25 stimuli of subject P1 are highlighted by the open symbols (individual trials) and bold black regression lines and regression results in the lower-right of each sub-panel. The listener was quite precise in localizing the azimuth components of the stimuli (evidenced from the high r^2 values, in combination with high gains and low biases), regardless the changes in NRI and ORI. The regression results for the other subjects are indicated by the green lines and were quite consistent. Despite the large variation in NRI and ORI, over a 36 dB range in both acoustic dimensions, regression lines were nearly optimal, with only a modest scatter of the data around the regression line (high r^2). The performance was slightly affected at the lowest intensities and largest positive contrasts only (bottom row of the stimulus matrix), as evidenced by an increased variability around the regression line.

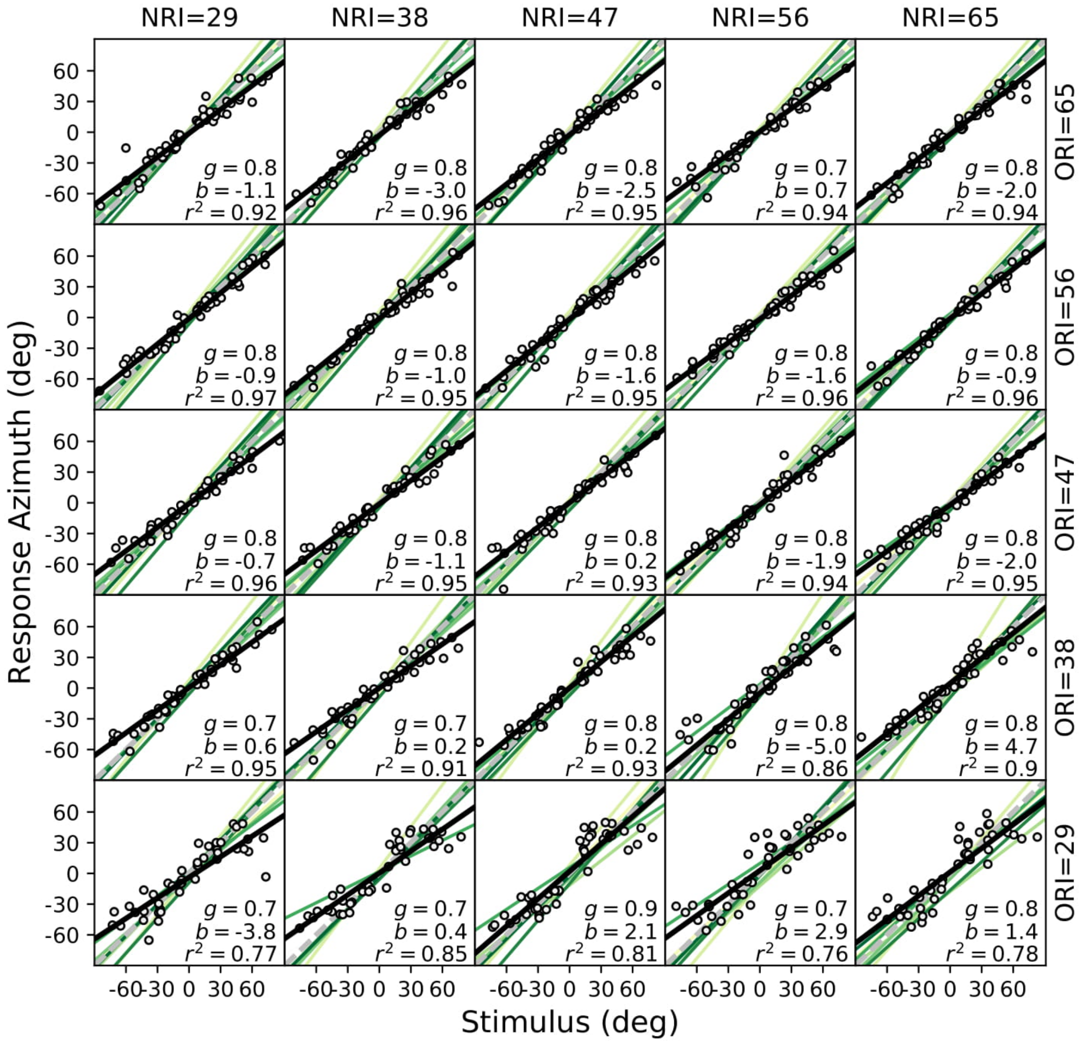

Figure S2. The azimuth stimulus-response plots for all 25 stimuli presented to subject P1. Dots denote the response data to single trials, and the solid black line denotes the linear regression line; the values in the bottom-right of each panels show the regression results (gain, g; bias in deg, b; variability, r^2). The linear regression lines for the other seven subjects are shown in green.

3. Average behavior in azimuth. To quantify a potential systematic azimuth dependency on spectral contrast and sound level, Figure S3 shows the spatial gains (left-hand column), biases (center column) and r^2 values (right-hand column), averaged across all subjects per stimulus in the same format as for the elevation responses in Figure 3. In contrast to the elevation response components, a nearly uniform pattern emerges for the response gain and bias, which did not vary significantly from stimulus to stimulus. There is only a small effect on the azimuth precision at the highest spectral contrasts and lowest sound levels (right-hand column). Responses are quite consistent across subjects.

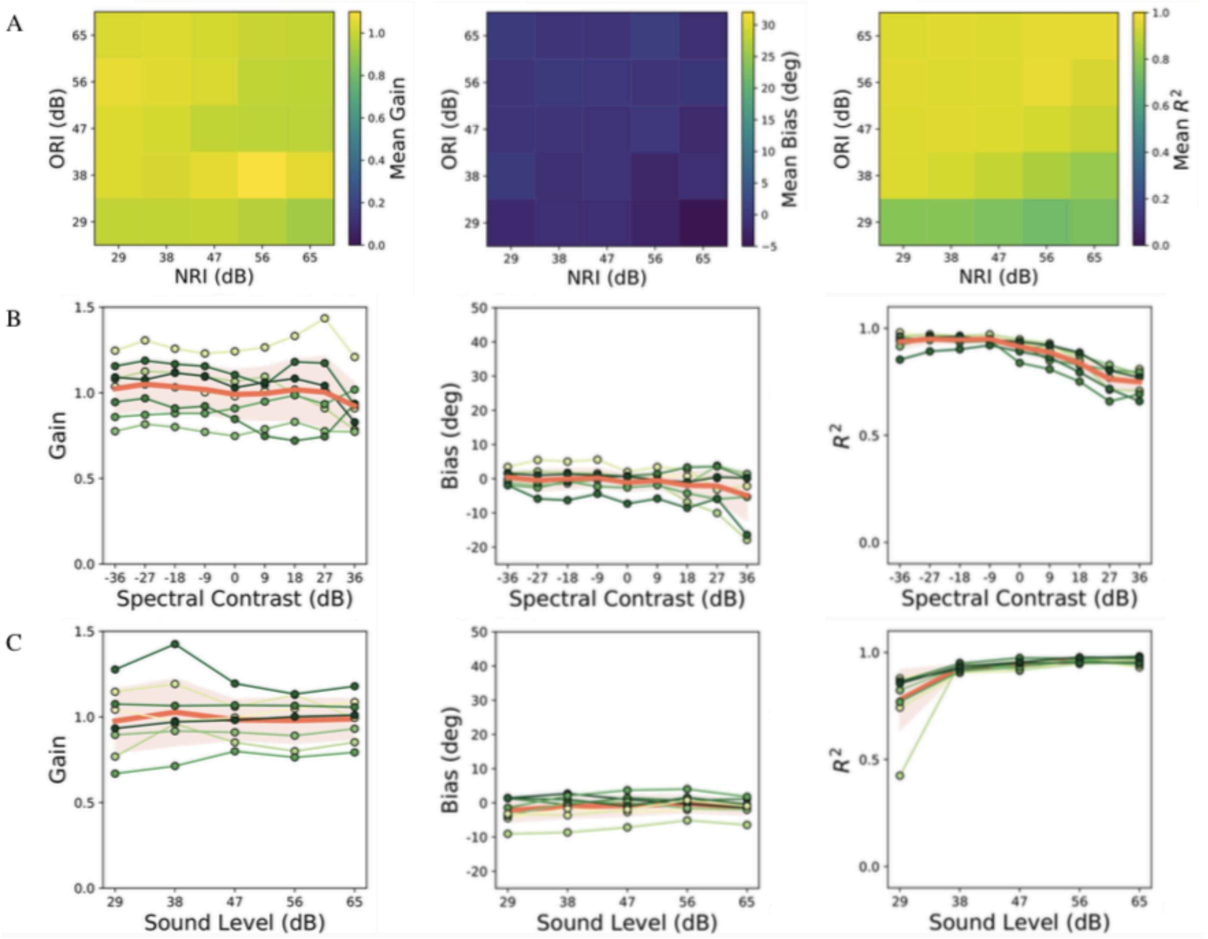

Figure S3. Average influence of the combination of NRI and ORI (A), the influence of spectral contrast (NRI-ORI) (B), and of absolute sound level (C), on the azimuth gain (left-hand column) and azimuth bias (center column) and azimuth precision (right-hand column) for all subjects. The thick solid orange line and shaded orange area correspond to the average and standard deviation for all subjects. Note that all subjects demonstrated very similar behaviour.

4. The Bayesian prediction for response gain. In our model we assume that in the first processing stage, the auditory system cross-correlates the (weighted) spectral sensory input, e.g., as measured by the auditory nerve and dorsal cochlear nucleus, with all learned and stored spectral pinna filters. The result leads, after rectification, to a likelihood function of potential target locations in elevation, ε, which depends on the current stimulus location, say at ε^*, here called $L(\varepsilon|\varepsilon^*)$. To ensure optimal localization, with minimal absolute localization errors (best accuracy), and minimum variability (best precision), the final estimation process involves the contribution of a spatial prior, $P(\varepsilon^*)$, which we assume is centered around straight ahead. Bayes' rule then transforms the likelihood function into a more precise posterior distribution, which specifies the probability to find the target at elevation ε^*, given the noisy sensory evidence and the spatial prior information:

$$\mathrm{POST}(\varepsilon^*|\varepsilon) \propto L(\varepsilon|\varepsilon^*) \cdot P(\varepsilon^*)$$

Multiplication of two Gaussian distributions again yields a Gaussian distribution, with a mean that lies between the two means of the original Gaussian distributions, and with a variance that is smaller than either of the two Gaussian distributions. Suppose that the prior and likelihood function can be described by the following two Gaussian distributions:

$$P(\varepsilon^*) = N_P \cdot \exp(-(\varepsilon^*)^2/(2\sigma_P^2)) \quad \text{and} \quad L(\varepsilon|\varepsilon^*) = N_L \cdot \exp(-(\varepsilon^*-\varepsilon)^2/(2\sigma_T^2))$$

where N_P and N_L are normalization constants, and σ_P and σ_T are the widths of the distributions. In that case, it follows that their product yields a posterior distribution for which the mean and variance are calculated as:

$$\mu_{POST} = \frac{\sigma_P^2}{\sigma_P^2 + \sigma_T^2} \cdot \varepsilon^* \quad \text{and} \quad \sigma_{POST}^2 = \frac{\sigma_P^2}{\sigma_P^2 + \sigma_T^2} \cdot \sigma_T^2$$

The optimal Bayesian response is found by taking the maximum of the posterior distribution (the Maximum-A-Posteriori, or MAP, estimate). Over the course of many trials, these maxima are found at the mean of the posterior. We thus define the optimal response gain as

$$g_{OPT} = \frac{\mu_{POST}}{\varepsilon^*} = \frac{1}{1 + \frac{\sigma_T^2}{\sigma_P^2}}$$

Note that in the absence of sensory noise, $\sigma_T \approx 0$, the response gain $g_{OPT} \approx 1$. When the noise is large, i.e., $\sigma_T \gg \sigma_P$, the response gain $g_{OPT} \downarrow 0$. The former condition is typically obtained for the azimuth response components, for which the binaural difference cues (per frequency channel) can be measured quite reliably, except at the poorest SNR's. The latter condition (low gain), however, can be readily obtained for the elevation response components under poor spectral conditions (at low SNR's (soft sounds), or for low and poor spectral resolution).

Other priors. Interestingly, our extended model in Figure 4 also accounts for the low response gains obtained for cases where the sensory evidence is actually very strong (e.g. when the 6-9 kHz notch

region is boosted; lower-right corner of the stimulus matrix in Figure 6), yet pointing at a particular, fixed elevation (a considerable upward bias for high positive spectral contrast stimuli). The reason for this counterintuitive behavior (at least in terms of the simple Bayesian framework described above) results from the fact that our model adopts *four* independent priors, instead of only one:

(i) the pinna prior: pinna filters are uncorrelated (i.e., they are unique for each elevation angle)
(ii) the prior on source spectra: natural source spectra do not correlate with any of the pinna filters.
(iii) the prior on frequency bands: some frequency bands are more informative for changes in elevation than others, and
(iv) the spatial prior: the expected distribution of potential target locations.

The low-gain/high-bias responses at high-positive spectral contrasts are thus caused by a strong dominance of the third prior, leading to a peak in the likelihood function that is unrelated to the actual stimulus location, but points consistently to upward stimulus positions.

5. Model simulations. To simulate the qualitative elevation response patterns of the subjects, as summarized in Figure 3A-C, with the model of Figure 4, we approximated human HRTFs by a canonical set of spectral shape functions (Figure S4A), whereby the elevation-independent ear-canal resonance was positioned at 2.5 kHz (modeled by a Gaussian spectral filter with a width of 1 kHz and an amplitude of +15 dB); the center of the spectral notch ran between 6 and 9 kHz in an elevation-dependent way (linear dependence, for simplicity), whereby the width of the notch decreased systematically with elevation; at high elevations (> 35 deg) a peak emerged in the HRTFs at 8 kHz that changed its center with increasing elevation towards lower frequencies (compare with Figure 1). See section **S6** for the details.

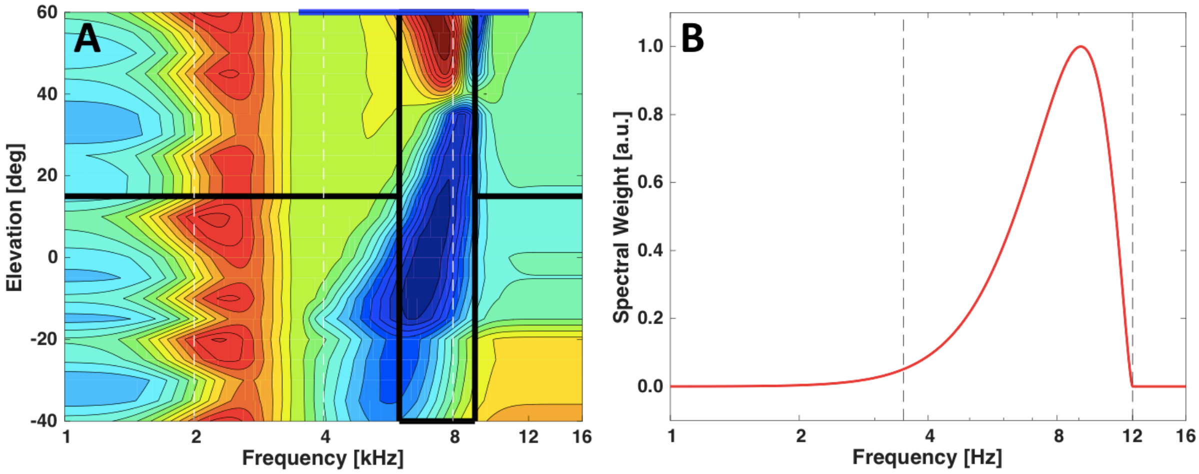

Figure S4 (A) Parameterized approximation of human HRTFs (cf. Figure 1) as used in the model simulations of Figure 5. **(B)** The spectral weighting function as used in the simulations.

Data availability. The experimental data can be obtained from the corresponding author upon reasonable request.

6. Matlab source code. The Matlab script, used to run the simulations.

```
function SimulatedHRTFs

%%%%%%.   Generation of the stylized HRTFs (modeled after the example HRTFs of Fig. 1)   %%%%%%%%%%

E=[-60:5:60];                     % elevation range
NE = length(E);                   % number of elevation angles
f=1:0.025:16;                     % frequency axis in kHz
f6 = find(f==6);                  % index of 6 kHz
f9 = find(f==9);                  % index of 9 kHz
                                  %   the spectral weighting function (between 3.5 - 12 kHz)
f35 = find(f==3.5);               % index of 3.5 kHz
f12 = find(f==12);                % index of 12 kHz
NF = length(f);                   % number of frequencies

H=zeros(NE,NF);                   % the HRTF matrtix

sc = 0.25;                        % width of the ear-canal resonance
fc = 2.5;                         % index of 2.5 kHz (location of the ear-canal resonance)

A=15; B=-15; C=15; D=5;           % scalings in dB (heights of peaks and depths of notches)
fo=find(f==3.5);
sel1 = f<3.5;                     % spectral range for the non-elevation cues
sel2 = f>3.5;                     %  spectral range for the notch

for n=1:NE.           %  calculate attenuation/amplification for each elevation angle in the different frequency regions (<3.5 and >3.5 kHz)
  argc = -(log(f)-log(fc)+0.05*randn(1,1)).^2/(2*sc^2);         % the ear-canal resonance; we add a bit of noise
  h1 = A*exp(argc).*sel1;
  h0=h1(fo-2);
  h1 = (h1-h0).*sel1;

  sn = 1.8 - 0.015*(E(n)+40);                          % the width of the notch decreases with elevation. (see Figure 1)
  sp = 1.0 - 0.05*(E(n)+40);                           % the width of the first peak decreases with elevation

  argn1 = -(f-7.0-0.038.*E(n)+0.05*randn(1,1)).^2/(2*sn^2);
  argp1 = -(f-4.0-0.038.*E(n)+0.05*randn(1,1)).^2/(2*sp^2);
  h2 = B*exp(argn1).*sel2;

  % ensure that h2 connects to h1 at 3.5 kHz
  h00 = h2(fo+1);
  h2 = (h2-h00).*sel2;

  h4 = D*exp(argp1);
  h04 = h4(fo+1);
  h4 = (h4-h04).*sel2;

  h3=zeros(1,NF);
    if E(n)>35.                                          % for high elevations
        sp = 0.8;                                        % width of the high-elevation peak in the HRTF (see Figure 1)
        argp = -(f-8.5+0.05.*(E(n)-35)+0.05*randn(1,1)).^2/(2*sp^2);
        h3 = C*exp(argp).*sel2;
  end

  H(n, :) = h1+h2+h3+h4;                        % the total HRTF across all frequency bands
end

%
%   Generate the normalized spectral weighting function, W(f)     (nonzero from 3.5 to 11 kHz)
%
a1=4.5; a2=1.5;      fo=3.5;    g=0.1;    fm=11;
sel  = (f >= g*fo);
sel2 = (f <= fm);
beta = (f-g*fo).^a1.*(fm-f).^a2;
beta = beta.*sel2;
W = beta.*sel/max(beta);
```

```
%             Test the model: run the simulated experiment
%
%              1. provide the notch-peak stimuli with varying contrast
%                 and present them at all elevations
%              2. For each sound, determine the (linear) stimulus-response relation:
%                 To determine the response, use the Bayesian idea:  Gaussian prior (straight ahead, (E=0, sigma 12 deg, or uniform)
%.                is multiplied with the (rectified) likelihood function (cross-correlation of the convolved spectral input with the HRTF)
%.                to give the posterior.
%                 The location of the maximum is the response elevation (MAP decision criterion on the posterior)
%
Xf=zeros(1,NF);

Prior = zeros(1,NE);
zero = find(E==0);
sp = 12;                                                    % about 40 deg width
for n=1:NE
   %    Prior(n) = 1/sqrt(2*pi*sp)*exp(-(n-zero)^2/(2*sp^2));      % Gaussian prior
        Prior(n)=1/NE;                                             % uniform prior (as used in the paper)
   end

Contrast = -36:4.5:36;                                      % NC = 11 contrasts, in dB
NC = length(Contrast);
RESP = zeros(NE,NC);                                        % the total response matrix for all elevations and each contrast

for k = 1 : NC                                              % outer loop across sounds
  Xf(f6:f9) = Contrast(k);                                  % the spectral-contrast stimulus
  Xf = Xf + randn(1,NF);                                    % add some noise

  for m = 1 : NE                                            % inner loop across elevations

     SfE = Xf + H(m,:);                                     % sensory spectrum for the sound at E(m) (add the dB's:  logarithmic scales)
     SWfE = W .* SfE;                                       % the weighted sensory spectrum
     C = zeros(1,NE);                                       % the correlation vector (all elevations)
     for n=1:NE
            c = corrcoef(H(n,1:f12), SWfE(1:f12));          % do the cross-correlation across all HRTFs
           C(n)= c(2,1);
        end
      sel = (C>0);
      LikeliHood = C .* sel;                                % rectification: the Likelihood function
     Posterior = Prior .* LikeliHood;                       % Bayes' rule
    RESP(m,k) = E(Posterior==max(Posterior));               % MAP (for a uniform prior, it's a maximum-likelihood estimation)
  end      % ELEVATION loop
end     % Contrast loop

%%%%    Plot the results and do the regressions     %%%%

figure(4); clf;   imagesc(RESP);  axis xy;
figure(5); clf;  hold on;                             % each contrast its own subplot
for n=1:NC
    subplot(4,4,n);        plot(E, RESP(:,n),'ko-');   axis([-60 60 -60 60]);
    c=corrcoef(E,RESP(:,n));                          % stimulus-response correlation
    Reg = polyfit (E, RESP(:,n)', 1);                 % linear regression on the responses; Reg contains the fit parameters.
    tmp = polyval(Reg, E);                            % plot the regression line
    s=sprintf('g=%2.2f  b=%2.2f  r2 = %3.3f' ,...
                   Reg(1),  Reg(2),  c(1,2)^2);  text(-35,55,s);
    gain(n) = Reg(1);                                 % slope of the stimulus-response relation
    bias(n) = Reg(2);                                 % bias
    R(n) = c(1,2)^2;                                  % r^2  (goodness of fit)
end

figure(6); clf; hold on;      plot(1:NC, gain, 's-r','Linewidth',2.5);
                              plot(1:NC, bias/50, 'o-b','Linewidth',2.5);    plot(1:NC, R, 'd-k','Linewidth',2.5);
xlabel('Spectral Contrast');    ylabel('Parameter');        set(gca,'linewidth',1.5);
set(gca,'xlim',[0.5 NC+.5],'xtick',1:NC,'xticklabel',Contrast, 'ylim',[-.2 1.2],'ytick',0:.2:1,'yticklabel',0:.2:1,'fontsize',16);
legend('Gain','Bias/50','r^2');    box on

return
```
